# Trends in mobile phone ownership, frequency of number changes, and implications for public health service delivery in Uganda, 2010–2020

**DOI:** 10.1038/s41598-025-10887-1

**Published:** 2025-07-11

**Authors:** Robert Ssekubugu, Ping Teresa Yeh, Hadijja Nakawooya, Victor Ssempijja, Godfrey Kigozi, Joseph Kagaayi, Fred Nalugoda, Anna Mia Ekström, Betty Nantume, David Serwadda, Philip Kreniske, Zangin Zeebari, Michelle A. Moffa, Larry W. Chang, Kate M. Grabowski, Fredrick Makumbi, Helena Nordenstedt

**Affiliations:** 1 Department of Global Public Health, Karolinska Institutet, Stockholm, Sweden; 2 Rakai Health Sciences Program, Kalisizo, Uganda; 3 Department of International Health, Johns Hopkins Bloomberg School of Public Health, Baltimore, MD USA; 4 Clinical Monitoring Research Program Directorate, Frederick National Laboratory for Cancer Research, Frederick, MD USA; 5 Department of Epidemiology and Biostatistics, Makerere University School of Public Health, Kampala, Uganda; 6 Department of Infectious Diseases, South General Hospital, Stockholm, Sweden; 7 Community Health and Social Sciences Department, Graduate School of Public Health and Health Policy, City University of New York (CUNY), New York, USA; 8 Jönköping University, Jönköping, Sweden; 9 Department of Epidemiology, Johns Hopkins Bloomberg School of Public Health, Baltimore, MD USA; 10 Department of Pathology, Johns Hopkins School of Medicine, Baltimore, MD USA; 11 Division of Infectious Diseases, Department of Medicine, Johns Hopkins School of Medicine, Baltimore, MD USA; 12 Department of Medicine and Infectious Diseases, Danderyd University Hospital, Stockholm, Sweden

**Keywords:** Mobile phone ownership, Mobile number changes, Surveillance, mHealth dynamics, Rural Uganda, Medical research, Epidemiology

## Abstract

Mobile phones significantly improve access to healthcare, public health services, and disease surveillance globally. However, challenges related to reachability and accessibility persist, especially when individuals change or drop telephone numbers affecting continuity in public health interventions such as HIV follow-ups and vaccine reminders. We explored trends in phone ownership, changes in mobile phone numbers, associated factors, and the time it takes to better understand how these might affect the ability of phone-based public health services to reach targeted recipients. We used data from the Rakai Community Cohort Study, a population-based prospective open cohort in rural Uganda. Between 2010 and 2020, data on phone ownership and individuals’ phone numbers were collected for six consecutive visits. We assessed trends in phone ownership using descriptive statistics. Factors associated with the number of times individuals changed their phone numbers were assessed using a Poisson multivariable regression model. We used Anderson Gill Cox proportional hazards regression to evaluate the time-to-change of phone numbers. In total 41,922 participants contributed 97,034 visits. A majority (61.8%) of participants owned a mobile phone at some point from 2010 to 2020. Phone ownership increased over the study period from 51.2% in 2010 to 68.2% in 2020 (*p* < 0.001). Phone ownership was lower among women participants (versus men; adjPR = 0.81; 95% CI 0.78–0.83) and younger persons < 25 years (versus ≥ 25 years; adjPR = 0.69; 95% CI 0.65–0.72), but there were no differences by HIV serostatus. The rate of change in phone numbers was significantly lower among women participants (adjusted prevalence ratio [adjIRR] = 0.88; 0.83–0.95) and those with secondary education or above (versus primary or none; adjIRR = 0.92; 95% CI 0.87–0.98). In contrast, it was higher among young persons aged 15-24-years old (versus 45 + years; adjIRR = 2.52; 95% CI 2.13–2.76), those living in lake Victoria fishing communities (versus trading centers, adjIRR = 1.28; 95% CI 1.17–1.40), persons with lower SES (versus higher SES; adjIRR:1.30; 95% CI 1.19–1.42), and persons living with HIV (versus HIV negative participants; adjIRR = 1.11; 95% CI 1.03–1.20). In this Ugandan cohort, mobile phone ownership increased over time, although by 2020 nearly 30% of the population still did not own a phone, and participants frequently changed phone numbers. Being a man, living with HIV, and lower socioeconomic status were all associated with changing phone numbers, a community peer system to maintain contact with these groups may be required to supplement phone-based initiatives.

**Supplementary Information:**

The online version contains supplementary material available at 10.1038/s41598-025-10887-1.

## Introduction

Mobile phones have become relatively widespread, with around 75% of the world’s population having access to a mobile phone^1^. Within the public health sector, the use of mobile phones targeting people with chronic non-communicable diseases has shown potential in LMICs^2^. Mobile health interventions associated with increasing healthy behaviors in the fields of diabetes, stroke, cardiovascular diseases, and maternal, newborn, and child health have demonstrated good health outcomes and treatment compliance^3–6^. In the area of HIV/AIDS, the use of mobile phone calls and reminder short message service (SMS) messages contributed extensively towards solving long-standing bottlenecks such as poor retention in HIV care^7^. Three studies that evaluated text messaging and phone counseling interventions reported improved biological outcomes^8^. An overview of systematic reviews about text messaging interventions for managing HIV and other chronic diseases found global evidence supporting the use of text messaging as a tool to improve HIV treatment adherence and appointment attendance^9^. Despite these promising successes, though, two systematic reviews of using mobile phones in HIV programming in LMICs have found equivocal evidence of effectiveness^7,10^.

In the context of surveillance—understood here as the ongoing, systematic collection, analysis, and interpretation of health data essential for planning, implementing, and evaluating public health practices—this process is closely integrated with the timely dissemination of such data to those who need it for informed decision-making^11^. The use of mobile phones in public health surveillance offers significant advantages for recruiting participants, particularly in geographically dispersed or hard-to-reach areas. Compared to face-to-face interviewing, mobile phone-based methods eliminate the need for interviewers to travel, saving both time and costs, and removing risks associated with fieldwork in dangerous or unstable areas such as flooded regions, conflict zones, or during public health emergencies^12,13^. Moreover, mobile phones enhance privacy, potentially improving data quality. Respondents may feel more comfortable sharing sensitive or personal information over the phone, reducing social desirability bias that often affects face-to-face interviews^13,14^. This is particularly valuable in research involving stigmatized health conditions or behaviors.

Despite these advantages, there are recruitment-related challenges. Systematic reviews have identified structural barriers, such as poor network coverage, limited access to electricity, and the dynamic nature of mobile phone usage in low-resource settings^15,16^. In Uganda, for example, there are nearly 29 million active mobile subscriptions—almost two lines per adult among the 17 million adult population. However, phone ownership is uneven: some individuals have multiple Subscriber Identity Module (SIM) cards or phones, while others have none, complicating equitable recruitment efforts^17^.

Maintaining consistent contact with participants after enrollment is critical for surveillance and research. However, follow-up via mobile phones faces persistent challenges. A key issue is phone number instability. In a tuberculosis study in Uganda, a quarter of urban participants changed their phone numbers during the study, leading to interruptions in communication and participation^18^. Similarly, a study on antiretroviral therapy adherence among young adults in Uganda found it common for participants to change phone lines without informing healthcare providers, compromising continuity in follow-up^19^. Such disruptions pose serious threats to large-scale public health studies, which depend on reliable communication to retain participants and collect longitudinal data. Loss to follow-up due to disconnected numbers can result in incomplete data and biased findings^20^. A contributing factor is the broader context of mobile phone use in Uganda and similar settings. People often change numbers due to affordability issues, phone theft, or privacy concerns. This reality makes it difficult for researchers to maintain stable communication channels.

One promising solution is suggested by a South African study highlighting the benefits of concurrent ownership of multiple phone lines^20^. This practice is quite common in some parts of Africa. Under this paradigm, since participants often use more than one active phone number, leveraging the collection of a participant’s multiple phone numbers could reduce the risk of lost contact. Researchers can continue communication through an alternate number even if one number becomes inactive, thus enhancing sustained participant accessibility throughout a study.

Determining the level of usefulness of mobile phone number registries is essential in supporting public health programming and research that uses mobile phone numbers to reach survey participants and program recipients. The purpose of this study is to use data from an established population-based cohort in south-central Uganda to (a) assess trends in phone ownership, rate of change of phone numbers, and possession of multiple phone numbers; (b) determine the rate and factors associated with changing phone numbers and if phone number changes may be associated with HIV status; and (c) estimate the time-to-change of phone numbers in a rural Ugandan population.

## Methods

### Study design and setting

Since 1994, the Rakai Health Sciences Program, through the Rakai Community Cohort Study (RCCS) in south-central Uganda, has been collecting and disseminating data on population trends including demographic characteristics, sexual behaviors, HIV risk factors, and HIV prevalence and incidence^21–24^. The RCCS also collects data on household structures and amenities, which is useful in estimating individuals’ and households’ socioeconomic status (SES)^25,26^. The RCCS has two major field components: (i) a census enumerating household membership and possessions and (ii) a survey a few weeks later involving same-sex behavioral interviews, vital measurements, and blood sample collection. The RCCS is an open, population-based cohort of people aged 15–49 years in 34 communities, 30 of which are inland communities that have been continuously surveyed since inception. Four lakeside fishing communities have been part of the cohort since 2010. Communities are considered rural if the primary activities of livelihood are largely agrarian in nature, urban if the activities are primarily related to trade and fishing communities if they are located by the lakeside and activities are primarily related to fishing^27^.

### Data source

Starting in 2010, the RCCS has collected data on mobile phone ownership and access (the ability to use a mobile phone among those who do not own one). These data have been collected for six RCCS census and survey visits from 2010 to 2020, with approximately 18–24 months between survey visits. Data on mobile phones are collected for every member of each household. The census data are collected at each household by trained and experienced enumerators. An adult (18 years and older) household member is asked to provide information about each household member. If no adult was present, another member or a neighbor provided the information, which is considered as a tentative record pending update should an adult household member be found during the survey. During census, the enumerator asks if the person owns a mobile phone (e.g., ‘Does [name] own a phone?‘). If the answer is yes, their phone number(s) are recorded, along with a follow-up question about how many lines and handsets they currently own. When more than one phone number is provided for a given individual, respondents are asked to provide all phone numbers in order of their usage, starting with the most frequently used number. Census enumerators are not provided with phone numbers obtained from prior household censuses (i.e. are blinded).

During the survey, conducted about one month after the census, all census data on households including phone numbers are checked to ensure accuracy and completeness. Additional data on individual participants’ educational level, age (confirmed with date of birth), marital status, occupation, sexual behavior, and utilization of HIV services is collected during the individual participant survey (which includes an interview and HIV testing services with pre and post-test counseling)^23^. As part of data management routines, all RCCS participants are automatically assigned to an SES quartile. The wealth index classification code was developed previously using a principal components analysis that considers possession of key household assets including radio, bicycle, motorcycle, car, latrine, and electricity, and the nature of the roof, floor, and walls^26,28^.

### RCCS participation rate

From the census, the RCCS obtains mobile phone access data for about 90% of all residents, as phone information can be provided either by the respondent or an informant. Of all eligible participants from the census, approximately 78% are present at the time of the survey, while the others are absent due to school, work, or visiting. The mean participation rate among all eligible individuals for the survey was 64%, and this rate remained relatively consistent across different survey visits (range 59–67%)^23^. A recent report shows that 95% of those present participate in the survey interview and contribute biological specimens for HIV testing^26^.

### Statistical analysis

We assessed changes in individuals’ phone numbers across six survey visits with descriptive statistics. Phone ownership was confirmed and defined based on self-report of owning a mobile phone at the time of the RCCS survey. Change of phone number was determined based on participants who reported phone ownership during at least two surveys and defined as incidents in which participants changed all their phone number(s) from the one(s) recorded at the previous RCCS visit. In the primary analysis, individuals who owned more than one number and changed only one (or some) line(s) but maintained the other number(s) were not considered as cases of phone number change in the primary analysis. A secondary analysis was performed to estimate the rate of change of at least one of the previously given phone numbers The rate of change and factors associated with phone number change were estimated using a Poisson multivariable regression model with generalized estimating equations (GEE) which accounts for repeated measures within individuals and adjusts for within subjects’ correlation and a logical correlation structure. In the model, we adjusted for sex, age group, education level, marital status, community type, socioeconomic status and HIV status. Findings are reported as incidence rate ratios (IRR) with 95% confidence intervals. The incidence of phone number changes was calculated as the number of changes per 100 person-years of follow-up. Person-time was measured from study entry to phone number change, loss to follow-up, or study end. Participants who missed a given visit but returned with a new number were treated as new entries; those with the same number continued their previous observation. To account for repeated phone number changes within the same individuals, we used the Andersen-Gill extension of the Cox proportional hazards model. This survival analysis method accounts for recurrent events and was used to evaluate the time to phone number change across study visits, from visit one to visit six, while adjusting for potential confounders—including age, socioeconomic status (SES), marital status, education, and type of community—to assess cumulative differences in survival functions by sex. Participants were excluded if they missed a survey visit, regardless of whether their phone number had been obtained from another household adult. They were re-enrolled upon resuming participation. Censoring was based on whether they had changed their phone number upon return; if not, they were reinstated and treated as continuous participants’ mobile number.

### Ethics

The RCCS was reviewed and approved by the Uganda Virus Research Institute Research Ethics Committee (UVRI-REC) and the Johns Hopkins Medical Institute Institutional Review Board and registered at the Uganda National Council for Science and Technology with clearance from the Office of the President of Uganda. Additional approvals were obtained from Karolinska Institutet to use RCCS data for this doctoral study. All the data for this study was collected, managed, and analyzed in Uganda. Three levels of informed consent were provided for RCCS procedures: (1) community consent obtained through dialogue meetings with local leaders and the community advisory board (CAB) prior to the start of each survey visit and oftentimes mid-way through the survey visit^29^, (2) verbal informed consent provided at the household census level by an adult household member or the informant, and (3) individual written informed consent obtained from all eligible and willing study participants. The data were anonymized by the data manager using computer-generated identifiers for each of the individuals. All study methods and procedures were performed in accordance with the relevant local and international guidelines and regulations for instance the Helsinki declaration, the principles in the Belmont report governing research involving humans^30–32^.

## Results

### Demographics and trends in phone ownership

A total of 41,922 participants contributed 97,034 person visits. Phone ownership (61.8%) is significantly higher among men (67.5%), adults aged 25 or older, and those with secondary education or higher (71.8%) (*p* < 0.001). Ownership also increases with SES, from 48.6% in the lowest group to 73.2% in the highest. Lower ownership is seen among youth aged 15–24 (44.9%), the never married (46.4%), and low-SES individuals. Nearly three-quarters (74.6%) of participants reporting more than one sexual partner in the past 12 months owned a mobile phone, compared to 58.1% who reported zero or one partner in the same period of follow-up (*p* < 0.001) (Table 1).

**Table 1:** Phone ownership patterns by participant characteristics, including demographics, socioeconomic status, and health indicators.

	Phone ownership		
	Yes	No	*p*-value
	*n* (row%)	*n* (row%)	
Overall	60,004 (61.8)	37,030 (38.2)	
Sex			
Men	30,129 (67.5)	14,490 (32.5)	< 0.001
Women	29,875 (57)	22,540 (43)	
Age group			
45+	4428 (70.6)	1840 (29.4)	< 0.001
34–44	17,201 (72.7)	6458 (27.3)	
25–34	23,044 (69.9)	9908 (30.1)	
15–24	15,331 (44.9)	18,824 (55.1)	
Education level			
Primary and below	34,784 (56.2)	27,117 (43.8)	< 0.001
Secondary and above	25,220 (71.8)	9913 (28.2)	
Marital status			
Married	37,513 (68.7)	17,103 (31.3)	< 0.001
Previously Married	10,114 (64.2)	5643 (35.8)	
Never married	12,377 (46.4)	14,284 (53.6)	
Community type			
Trading community	24,089 (66.9)	11,912 (33.1)	< 0.001
Agrarian community	23,874 (59.3)	16,369 (40.7)	
Fish landing site	12,041 (57.9)	8749 (42.1)	
Socioeconomic status			
Highest	18,882 (73.2)	6907 (26.8)	< 0.001
High middle	15,764 (68.1)	7376 (31.9)	
Low-Middle	13,308 (57.8)	9704 (42.2)	
Low	11,670 (48.6)	12,362 (51.4)	
Missing	380 (35.8)	681 (64.2)	
Number of sexual partners in past 12 months			
0 to 1 partner	43,548 (58.1)	31,436 (41.9)	< 0.001
More than 1 partner	16,456 (74.6)	5594 (25.4)	
HIV status			
Negative	48,537 (61.5)	30,329 (38.5)	< 0.001
Positive	11,467 (63.1)	6701 (36.9)	

**Fig. 1:**
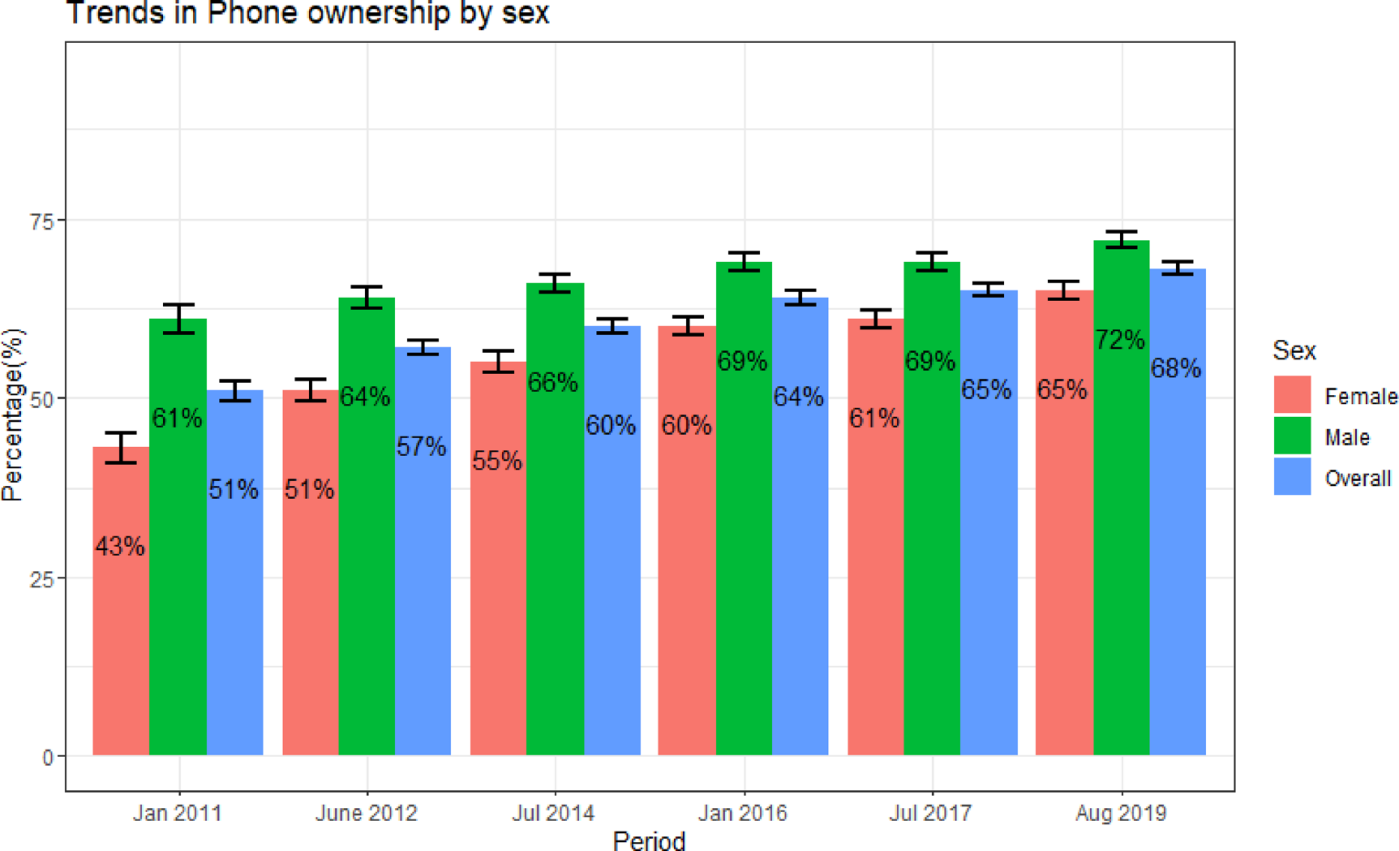
Trends in mobile phone ownership (overall and by sex) in the Rakai CommunityCohort Study population (2010-2020). The period is the median time point of a surveyvisit.

Phone ownership increased by 33.2% over the study period, from 51.2% in 2010 to 68.2% in 2020 (*p* < 0.001) (Fig. 1). Differences in mobile phone ownership by sex reduced over the study period from 34.6% in 2010 to 8.9% in 2020 (Fig. 1). Lower levels of phone ownership were observed among women vs. men (adjPR = 0.81, 95% CI 0.78–0.83), lowest SES vs. high SES (adjPR = 0.78, 95% CI: 0.76–0.81), and younger vs. older (adjPR = 0.69, 95% CI 0.65–0.72) participants (Table 2). There was no difference in phone ownership by HIV status (adjPR = 1.01, 95% CI 0.97–1.05).

**Table 2:** Unadjusted and adjusted generalized Estimation equation model for phone ownership 2010–2020.

Characteristics	Unadjusted		Adjusted	
	PR (95% CI)	*p*-value	PR (95% CI)	*p*-value
Sex				
Men	1		1	
Women	0.85 (0.83–0.88)	< 0.001	0.81 (0.78–0.83)	< 0.001
Age group				
45+	1		1	
34–44	1.00 (0.96–1.04)	0.935	1.00 (0.96–1.05)	0.838
25–34	0.93 (0.89–0.97)	0.001	0.94 (0.90–0.98)	0.004
15–24	0.65 (0.62–0.68)	< 0.001	0.69 (0.65–0.72)	< 0.001
Education level				
Primary and below	1		1	
Secondary and above	1.25 (1.22–1.29)	< 0.001	1.28 (1.25–1.31)	< 0.001
Marital status				
Married	1		1	
Previously Married	0.97 (0.94-1.00)	0.037	1.00 (0.97–1.03)	0.815
Never married	0.73 (0.70–0.75)	< 0.001	0.81 (0.78–0.84)	< 0.001
Community type				
Trading community	1		1	
Agrarian community	0.90 (0.87–0.93)	< 0.001	0.94 (0.91–0.97)	< 0.001
Fish landing site	0.88 (0.84–0.91)	< 0.001	0.96 (0.93-1.00)	0.081
Socioeconomic status				
Highest	1		1	
High-middle	0.95 (0.93–0.98)	< 0.001	0.96 (0.94–0.99)	0.003
Low–middle	0.88 (0.86–0.90)	< 0.001	0.89 (0.86–0.91)	< 0.001
Low	0.79 (0.76–0.81)	< 0.001	0.78 (0.76–0.81)	< 0.001
Missing	0.66 (0.59–0.74)	< 0.001	0.63 (0.56–0.70)	< 0.001
HIV status				
Negative	1		1	
Positive	1.03 (0.99–1.07)	0.105	1.01 (0.97–1.05)	0.6

### Possession of multiple lines

The number of individuals with multiple phone numbers increased from 1.9% in January 2011 to 7.2% in August 2019 (*p* < 0.01). Overall, women were less likely to have multiple phone lines compared to men (adjPR = 0.61, 95% CI 0.55–0.68), and those aged 15–24 continued to have the lowest likelihood of multiple line ownership (adj PR = 0.36, 95% CI 0.29–0.46) (Table 3). Lower SES was associated with a lower likelihood of multiple-line ownership. Individuals with secondary education and above were more likely to have multiple phone lines compared to those with primary education and below (adjPR = 1.64, 95% CI 1.48–1.82).

**Table 3:** Unadjusted and adjusted generalized Estimation equation model for multiple line ownership.

Characteristics	Unadjusted		Adjusted	
	PR (95% CI)	*p*-value	PR (95% CI)	*p*-value
Sex				
Men	1		1	
Women	0.67 (0.60–0.74)	< 0.001	0.61 (0.55–0.68)	< 0.001
Age group				
45+	1		1	
34–44	0.85 (0.72-1.00)	0.052	0.85 (0.72-1.00)	0.057
25–34	0.75 (0.64–0.89)	0.001	0.71 (0.60–0.84)	< 0.001
15–24	0.41 (0.33–0.51)	< 0.001	0.36 (0.29–0.46)	< 0.001
Education level				
Primary and below	1		1	
Secondary and above	1.58 (1.43–1.75)	< 0.001	1.64 (1.48–1.82)	< 0.001
Marital status				
Married	1		1	
Previously married	0.95 (0.83–1.08)	0.427	1.16 (1.01–1.33)	0.041
Never married	0.72 (0.62–0.84)	< 0.001	1.00 (0.84–1.19)	0.972
Community type				
Trading community	1		1	
Agrarian community	0.73 (0.65–0.82)	< 0.001	0.77 (0.69–0.87)	< 0.001
Fish landing site	1.11 (0.97–1.26)	0.119	1.49 (1.29–1.72)	< 0.001
Socioeconomic status				
Highest	1		1	
High–middle	0.87 (0.77–0.98)	0.023	0.89 (0.79-1.00)	0.051
Low–middle	0.71 (0.62–0.81)	< 0.001	0.72 (0.63–0.83)	< 0.001
Low	0.68 (0.59–0.80)	< 0.001	0.62 (0.52–0.73)	< 0.001
Missing	0.62 (0.29–1.29)	0.197	0.46 (0.22–0.95)	0.037
HIV status				
Negative	1		1	
Positive	0.88 (0.77–1.01)	0.061	0.85 (0.74–0.98)	0.025

Covariates included in the adjusted analysis were sex, age group, education level, marital status, community type, socioeconomic status, and HIV status.

### Non-possession of mobile telephones

Women were 48% more likely to report non-phone ownership compared to men (adjPR = 1.48, 95% CI 11.43–1.54), never married individuals were more likely to report non-phone ownership compared to married individuals (adjPR = 1.41, 95% CI 1.36–1.47). As SES decreased from “Highest” to “Low”, the likelihood of non-phone ownership increased significantly. Those with secondary education and above were less likely to report non-phone ownership compared to those with primary education and below (adjPR = 0.63, 95% CI 0.60–0.65).

### Persistent and rising phone number change over a decade

**Fig. 2:**
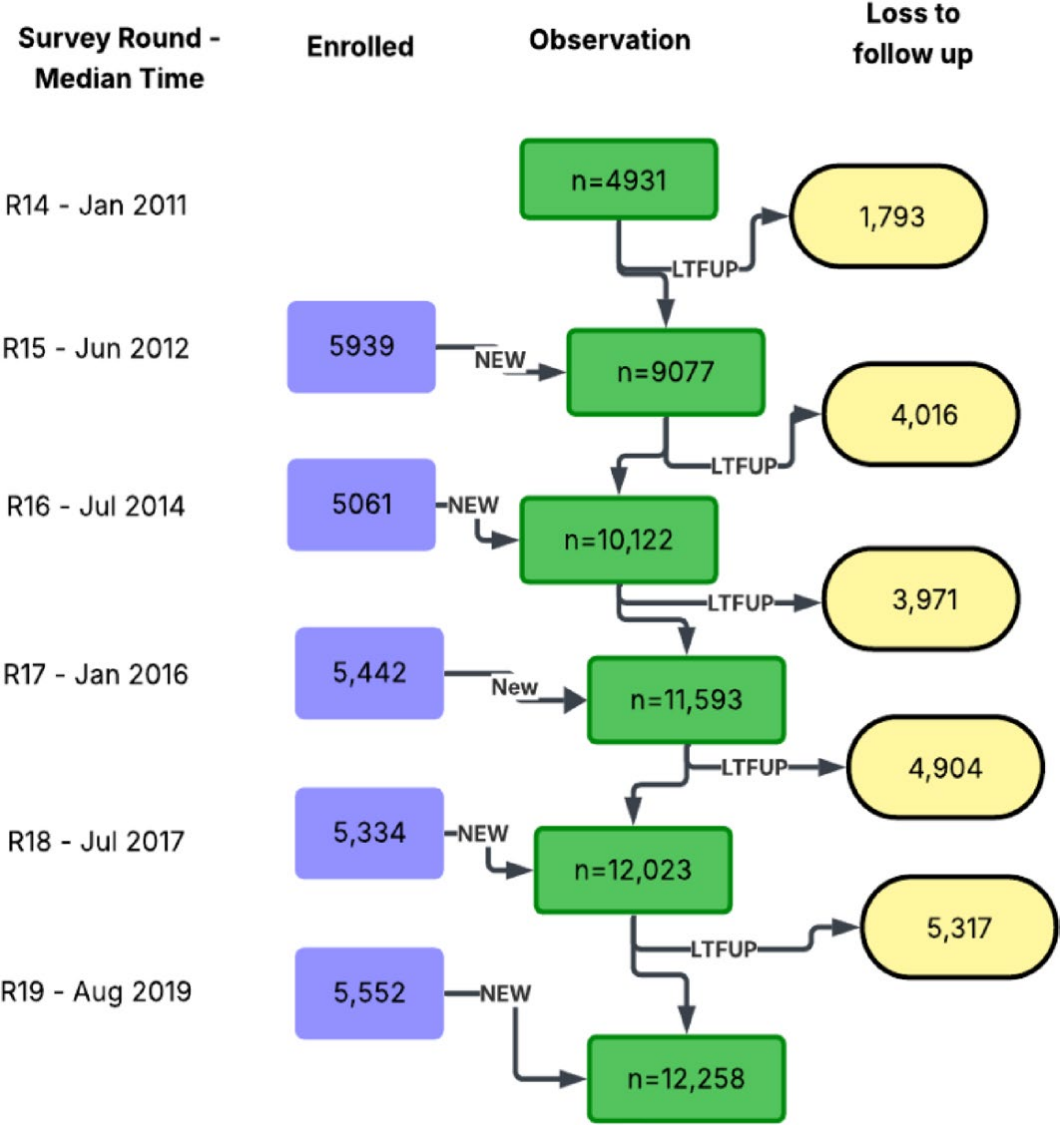
Depicts participant retention, continuous enrollment (open cohort), and loss to follow-up (LTFUP). Attrition primarily resulted from outmigration, aging out of the cohort, absence during surveys, discontinuation of phone service, and refusal to participate.

The absolute percentage of participants who changed their phone number(s) between two consecutive survey periods increased steadily from 26.7% (2011–2013), 30.3% (2013–2015), 29.8% (2015–2016), 32.6% (2016–2018), to 34.5% (2018–2020) (Table 4). The average total proportion of participants who switched their phone lines between consecutive survey visits was 31.4%. The number of participants owning a phone per visit rose from 4931 to 12,258, as depicted in Fig. 2.

**Table 4:** Phone ownership and change of telephone numbers among RCCS participants by survey visit.

Survey median date (range)—visit number	Present for the survey and owns a phone	Owns a phone and followed up at the next visit	Changed ALL telephone number in between visits	Total person-years
	No./total no. (%)	No./total no. (%)	No. (%)	
Jan 2011 (Jan 2010–June 2011)—R014	4931/9639 (51.2%)	0/4931 (0)		
June 2012 (Aug 2011–May 2013)—R015	9077/15,886 (57.1%)	3138/9077 (34.6%)	837 (26.7%)	4366.3
Jul 2014 (Jul 2013–Jan 2015)—R016	10,122/16,870 (60.0%)	5061/10,122 (50.1%)	1535 (30.3%)	8427.3
Jan 2016 (Jan 2015– Sept 2016)—R017	11,593/18,105 (64.0%)	6151/11,593 (53.1%)	1835 (29.8%)	8378.0
Jul 2017 (Oct 2016–May 2018)—R018	12,023/18,562 (64.8%)	6689/12,023 (55.6%)	2179 (32.6%)	9476.2
Aug 2019 (Jun 2018–Nov 2020)—R019	12,258/17,972 (68.2%)	6706/12,258 (54.7%)	2315 (34.5%)	10,805.3
Total	60,004/97,034 (61.8%)	27,745/60,004 (46.2%)	8701 (31.4%)	41,453.1

The incidence of phone number change was consistently high across the ten-year period, ranging from 15 to 19 per 100 person-years (Table S1). The rate of changing at least one phone number increased alongside rising phone ownership, from 36.4% at the first follow-up in June 2012 to 73.5% at the last follow-up in August 2019 (Table S2).

### Risk factors for changing mobile phone numbers

During the study period, relative to men, women were less likely to change their mobile phone number (adjIRR = 0.88, 95% CI 0.8–0.95) (Table 5). Relative to older participants (45 years or older), younger participants were more likely to change their phone numbers, and the likelihood to change reduced with increasing age (adjIRR = 2.42, 95% CI 2.13–2.76) for 15-24-year-olds; (adjIRR = 1.53, 95% CI 1.37–1.71) for 25-34-year-olds; (adjIRR = 1.16, 95% CI 1.04–1.29) for 35–44-year-olds. Participants with at least secondary education had an 8% reduced risk of changing their phone numbers compared to those with primary education or less (*p* = 0.003). Relative to married participants, those who had never married were slightly more likely to change their phone number (adj IRR = 1.08, 95% CI 1.00–1.16), and there was no significant difference with those previously married (adjIRR = 1.04, 95% CI 0.90–1.11).

**Table 5:** Unadjusted and adjusted generalized estimation equation model of the incidence of phone number change.

Characteristics	Unadjusted		Adjusted	
	IRR (95% CI)	*p*-value	IRR (95% CI)	*p*-value
Sex				
Men	1		1	
Women	0.81 (0.77–0.87)	< 0.001	0.88 (0.83–0.95)	< 0.001
Age group				
45+	1		1	
35–44	1.15 (1.03–1.29)	0.010	1.16 (1.04–1.29)	0.008
25–34	1.53 (1.37–1.71)	< 0.001	1.52 (1.37–1.70)	< 0.001
15–24	2.52 (2.23–2.84)	< 0.001	2.42 (2.13–2.76)	< 0.001
Education level				
Primary and below	1		1	
Secondary and above	0.88 (0.83–0.94)	< 0.001	0.92 (0.87–0.98)	0.010
Marital status				
Married	1		1	
Previously Married	1.06 (0.98–1.15)	0.127	1.06 (0.98–1.15)	0.150
Never married	1.42 (1.30–1.55)	< 0.001	1.08 (0.98–1.18)	0.125
Community type				
Trading community	1		1	
Agrarian community	1.00 (0.94–1.07)	0.929	0.97 (0.90–1.04)	0.366
Fish landing site	1.53 (1.41–1.66)	< 0.001	1.28 (1.17–1.40)	< 0.001
Socioeconomic status				
Highest	1		1	
High middle	1.20 (1.12–1.29)	< 0.001	1.13 (1.06–1.22)	0.001
Low middle	1.21 (1.12–1.30)	< 0.001	1.12 (1.04–1.21)	0.004
Low	1.52 (1.40–1.65)	< 0.001	1.30 (1.19–1.42)	< 0.001
Missing	1.17 (0.76–1.81)	0.467	0.91 (0.59–1.39)	0.651
Number of sexual partners in past 12 months				
0 to 1 partner	1		1	
More than 1 partner	1.17 (1.10–1.24)	< 0.001	1.04 (0.97–1.11)	0.282
HIV status				
Negative	1		1	
Positive	1.09 (1.01–1.18)	0.020	1.11 (1.03–1.20)	0.010

Compared to residents in trading communities, participants residing in fishing communities were more likely to change their phone numbers during the study (adjIRR = 1.28, 95% CI 1.17–1.40). This difference was not observed with participants in agrarian communities (adjIRR = 0.97, 95%; CI 0.90–1.04). People living with HIV were more likely to change their phone number (adjIRR = 1.11, 95% CI 1.03–1.20) relative to people without HIV. We did not observe a significant difference in number change between participants reporting more than one sexual partner in the past 12 months relative to those reporting zero or one partner in the same period (adjIRR = 1.04, 95% CI 0.97-1:11). Relative to those in the highest SES quartile, participants in the lowest SES quartile were more likely to have changed their phone numbers at the subsequent survey visit and the risk of changing the phone number reduced with increasing SES (adjIRR = 1.30, 95% CI 1.19–1.42) for low SES; (adjIRR = 1.12, 95% CI 1.04–1.21) for low-middle SES; (adjIRR = 1.13, 95% CI 1.06–1.22) for high-middle SES.

### Time to change personal phone numbers

**Fig. 3:**
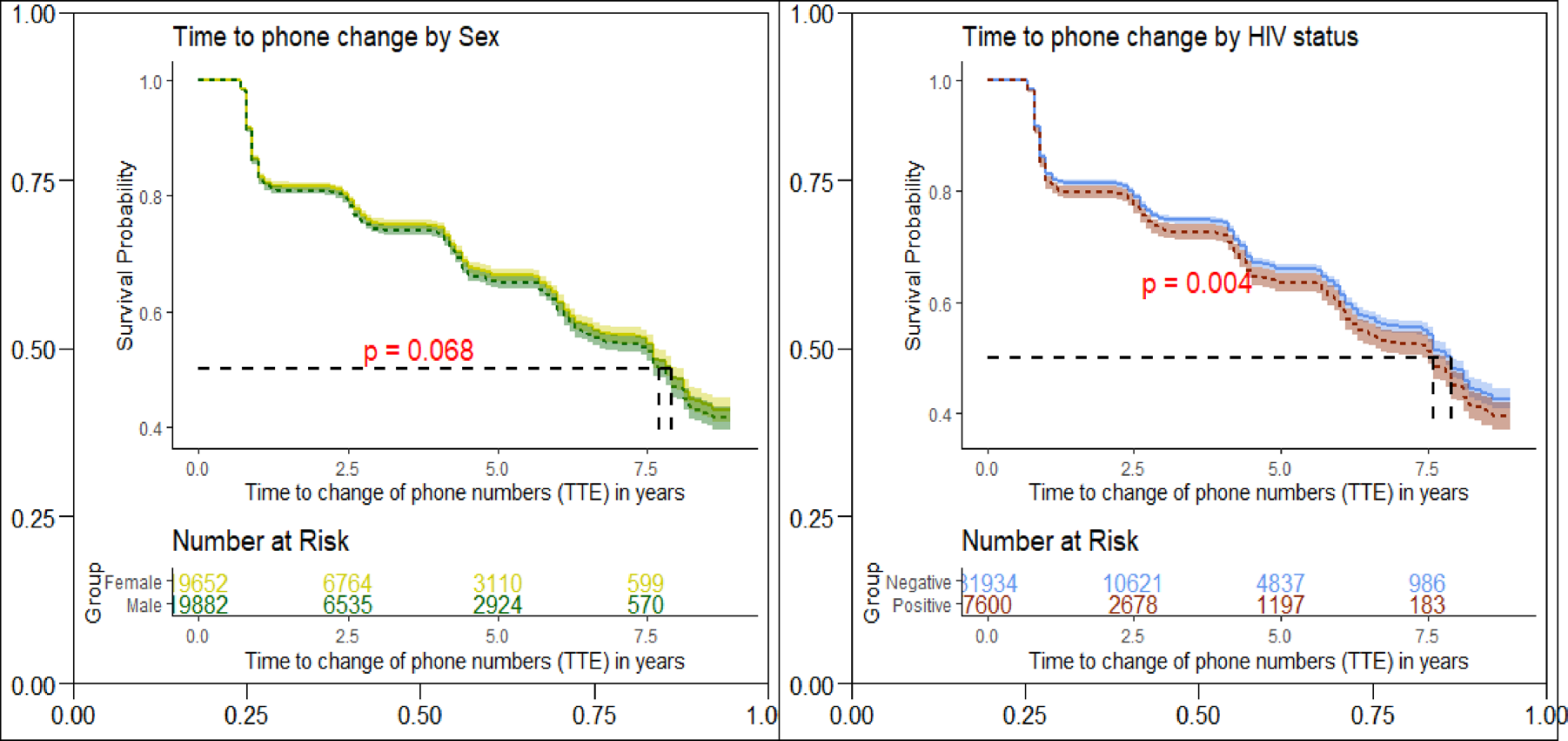
Time to phone change by sex (left panel) and HIV status (right panel) adjusted for other covariates.

Figure 3 shows the adjusted survival curves for the time-to-change of phone numbers, disaggregated by sex and by HIV status. Overall, 50% of participants changed their phone number(s) within 7.5 years. Among all the people followed at least once, only 50% of phone owners never changed their phone number(s) during the observation period. We did not observe a significant change by sex (*p* = 0.068), however, there was a significant difference by HIV status (*p* = 0.004). Among those surveyed between Jan 2010 and Jun 2011 (visit 14), 26.7% of the participants changed their phone lines (28.3% and 24.9% for men and women respectively) at the next visit. The rate of telephone number change increased with subsequent visits; 34.5% of those seen at visit 18 had changed their telephone lines by visit 19 (Table 6).

**Table 6:** Changed mobile phone numbers between visits by sex and HIV status.

Survey median date (range)—visit number	Changed mobile phone numbers between visits			
	Sex		HIV status	
	Men	Women	Negative	Positive
Jan 2011 (Jan 2010–June 2011)—R014				
June 2012 (Aug 2011– May 2013)—R015	469/1658 (28.3%)	368/1480 (24.9%)	708/2650 (26.7%)	129/488 (26.4%)
Jul 2014 (Jul 2013– Jan 2015)—R016	853/2636 (32.4%)	682/2425 (28.1%)	1187/4020 (29.5%)	348/1041 (33.4%)
Jan 2016 (Jan 2015–Sept 2016)—R017	1017/3148 (32.3%)	818/3003 (27.2%)	1446/4909 (29.5%)	389/1242 (31.3%)
Jul 2017 (Oct 2016–May 2018)—R018	1172/3307 (35.4%)	1007/3382 (29.8%)	1726/5354 (32.2%)	453/1335 (33.9%)
Aug 2019 (Jun 2018–Nov 2020)—R019	1231/3262 (37.7%)	1084/3444 (31.5%)	1858/5396 (34.4%)	457/1310 (34.9%)
Total	4742/14,011 (33.8%)	3959/13,734 (28.8%)	6925/22,329 (31%)	1776/5416 (32.8%)

## Discussion

In this open cohort of 27,745 participants in south-central Uganda, we observed a growing trend in mobile phone ownership from 51.2–68.2% over the 10-year study duration up to 2020. This is consistent with what has been reported in Uganda and other sub-Saharan African settings^33–35^. This growth in phone ownership was true for both sexes, but the gender disparity in mobile phone ownership reduced during the study period from a 33.9% difference in 2010 to an 8.9% difference in 2020. This progress demonstrates progress towards closing the gender gap in mobile phone ownership which may help address selection bias on phone surveys in rural sub-Saharan African settings^36,37^. Between 30–40% of the members in the study community did not own mobile phones by the end of the analysis period This level of non-phone ownership is consistent with the broader population in Uganda^35^. Most individuals without mobile phones are women, adolescents, younger adults, and people in the lowest SES groups. Multiple studies, including the Mobile Gender Gap Report in 2023^38^, confirm this pattern. This suggests that certain demographics are more likely to be excluded from mobile phone-based public health and development programs. The data underscores the significance of making basic and affordable mobile phones accessible to these marginalized communities. Affordable devices have the potential to bridge the digital divide, enabling broader access to critical services, including healthcare information. While the cost of mobile phone devices is generally becoming more affordable, some studies in Uganda suggest that affordability challenges persist. These challenges include the cost of telephone handsets, airtime (calling credit), and battery charging fees—particularly for households not connected to the national electricity grid”^39^.

We also observed that the proportion of individuals with more than one phone line increased over the study period. The Uganda Communications Commission estimates that there are thirty-three million active SIM cards, but many of these are owned through multiple subscriptions with one person having more than one line^40^. One study in South Africa associated multiple phone number ownership with improved reach-ability^41^. The ownership of multiple telephone lines can likely be explained by the variability of mobile network services in different locations. Network providers have varying levels of coverage and signal strength in different geographies^42^. To ensure reliable connectivity, individuals may choose to have multiple phone lines from different providers to have better coverage wherever they go. Cost is another important consideration for individuals. Making calls across networks is sometimes more expensive relative to calls within the same network due to different pricing plans, tariffs, or interconnection fees charged by network operators. To minimize costs, users choose to have multiple phone numbers to match the different networks of their frequent call recipients. The larger telephone service providers are also involved in phone-based money transfer and mobile banking services popularly known as mobile money services, cost of transferring money on the same network might be lower than transferring money across different networks^43,44^. The introduction of mobile banking has indeed been a significant innovation in addressing financial inclusion challenges in low- and middle-income countries. It has the potential to reach more people faster and at a lower cost compared to traditional banking infrastructure making mobile phone ownership increasingly more attractive^45–47^. There are still substantial challenges and disparities in reaching certain demographics, particularly women and individuals with lower SES^48^. This is consistent with our study results where this same demographic represents those most likely not to own phones and if they own, they do not own multiple phone lines.

About 30% of participants changed their mobile phone numbers between two survey visits (every 18–24 months). A tuberculosis study that took place in Kampala, Uganda, similarly reported that 26% (*n* = 145) of participants changed phone contacts during the study, and of those 16 (37%) changed within 6 months^18,49^. Such high levels of phone number change should be considered when planning for phone-based surveys, surveillance efforts, and other public health programs using stored phone contact databases. One potential cause of changes in phone numbers is phone loss due to frequent phone thefts in Uganda^49–51^. Compared to their men counterparts, women were less likely to change their phone numbers. This could be because men are more likely to go to areas such as major cities or trading zones where phone thefts are more common^52^.

Younger participants (15–24 years old) were more than twice as likely to change their phone number compared to those aged 45 or older. Phone-based surveys that sample from stored phone contact databases are likely to under-represent adolescents and younger adults. However, in other phone survey recruitment models, such as random digit dialing, younger respondents were more likely to participate, leading to their overrepresentation in the study sample as was reported by a Ghana study^53^. Given the higher rates of phone number changes among younger individuals in this setting, oversampling this population may help mitigate attrition. However, since our findings indicate that individuals who frequently change phone numbers differ systematically from those who do not, additional measures including analytic strategies may be needed to address potential bias resulting from this differential attrition^54,55^. The same is true for persons living with HIV/AIDS, who have an 18% increased likelihood of changing phone numbers relative to people without HIV. This could be explained by the high rates of phone change in high-risk HIV populations and individual, such as people residing in fishing communities^33^. The risk of phone loss is higher in fishing villages, possibly due to thefts or phones falling in water given the nature of work and the environment in the fishing areas. When a phone is lost, the process of acquiring a replacement of the same number requires owning a national ID for Ugandans. The prevalence of ownership of national IDs is lower in this region relative to other parts of the country^56^. The challenges related to the registration of previously owned mobile phone lines and how they lead to changes in phone line ownership have been reported in another study conducted in South Africa^57^. Therefore, changing or obtaining a new phone line is sometimes preferable to replacing the current number^58^, since any person family or friend with a national ID can assist in acquiring a new line.

Given that people living with HIV are more likely to change phone numbers relative to people without HIV, phone surveys sampling from stored phone databases may be more likely to miss people living with HIV, which could under-represent this population. Factors associated with HIV risk such as being a man, living in fishing communities, being younger, and reporting multiple sexual partners in the past 12 months are linked to a higher likelihood of changing a phone number sooner. This is an important consideration for HIV response efforts that propose using phone-based interventions. In a recent multicounty study, demonstrated that mobile phone connectivity had a positive impact of mobile-based HIV prevention schemes by showing through large-scale analysis that better mobile network access is a powerful tool to spread reproductive health knowledge and increase HIV awareness^33,59^. In the context of HIV services, continuity of access to a known phone line facilitated peer counselling and access to information on contemporary HIV services, treatment adherence more so, in the context of HIV and subsistence abuse^60,61^. While the use of mobile phones is generally acceptable in HIV care and response, there are mixed results for and against the use mobile phones in HIV response mostly on treatment adherence and/awareness calling for generation of more contextual evidence^62–64^.

Half of the participants changed their phone numbers by approximately 6 years of follow up. This is consistent with what has been reported in a study from South Africa which found only 50% of the study participants were reachable after 4.6 years (standard deviation 0.4 years)^41^. The South Africa study assessed levels of reachability using mobile phones^41^. Within a period of close to 1 year, 71% (141/200) could be reached via their most updated mobile phone contact (study I). That reach-ability rate reduced to 50% (100/200) as the period increased to 4.5 years (study II). With a total of 915 telephone numbers called for 400 participants, a 60% (241/400) reachability rate was reported during 3 rounds of calling. This study also highlights a critical aspect of multiple phone numbers per individual;^41^ a practice that is common in many African countries. It appears the more cellular service providers there are the more phone numbers most people will possess at a given time. From this study, the more telephone numbers a participant possesses the higher the odds of being reached later^41^. In another South African study, participants who at the time of the study owned a mobile phone reported high mobile phone turnover due to theft or loss 39% (*n* = 94) and/or damage 28% (*n* = 68)^65^. If replacement mechanisms for the old line are cumbersome, users will prefer to share or acquire a different phone number which might seem to be the case for Uganda currently.

This analysis had several limitations. Our data collection intervals span 18 to 24 months, and as a result, this analysis would benefit from an estimate of the annual phone change rate, which we are unable to calculate with the available data. We did not interview everyone to ascertain their current phone number(s). This analysis may be limited to mobile telephone ownership and does not account for access. However, it is important to recognize that some individuals may not own personal phone numbers or may experience frequent changes to their numbers, yet they can still be consistently and successfully reached through third-party (like a household contact or peer) phone lines.

In conclusion, in this Ugandan cohort, mobile phone ownership increased over time, yet by 2020, nearly 30% of participants still did not own a phone. Phone number instability was common, with about 50% of participants changing their phone lines within six years of follow-up. Changing phone numbers was associated with being a man, living with HIV, and having lower socioeconomic status. Although mobile phone ownership is on the rise among both sexes, frequent changes in phone numbers remain a challenge.

## Electronic supplementary material

Below is the link to the electronic supplementary material.

Supplementary Material 1

## Data Availability

Data beyond what is presented in this manuscript is available upon reasonable request to the corresponding author.

## Abbreviations

Adj IRR: Adjusted incidence rate ratio
Adj PR: Adjusted prevalence ratio
CI: Confidence interval
LMIC: Low and middle-income countries
PLWHA: People living with HIV/AIDS
RCCS: Rakai Community Cohort Study
SES: Socio-economic status
SIM: Subscriber Identify Module
