# Supplementary material for: Trends in mobile phone ownership, frequency of number changes, and implications for public health service delivery in Uganda, 2010–2020
Source: Sci Rep. 2025 Jul 11;15:25076. doi: 10.1038/s41598-025-10887-1 (PMC12254472; doi:10.1038/s41598-025-10887-1)
Supplement: Supplementary file 1 — Supplementary Material 1 [file 41598_2025_10887_MOESM1_ESM.docx]

**SUPPLIMENTARY TABLES**

**Table S1: Incidence of Phone Number Change by Survey Round over the 10-Year Period**

|  | **Failures (changed phones)** | **Person years** | **Incidence per 100 person years** |
| --- | --- | --- | --- |
| **Survey median date (range) - visit number** |  |  |  |
| Jan 2011 (Jan 2010 - June 2011) – R014 |  |  |  |
| June 2012 (Aug 2011 – May 2013) - R015 | 837 | 4371.8 | 19.14(17.89-20.49) |
| Jul 2014 (Jul 2013 – Jan 2015) - R016 | 1535 | 9805.4 | 15.65(14.89-16.46) |
| Jan 2016 (Jan 2015 – Sept 2016) - R017 | 1835 | 11343.9 | 16.18(15.45-16.93) |
| Jul 2017 (Oct 2016 – May 2018) – R018 | 2179 | 12210.3 | 17.84(17.11-18.61) |
| Aug 2019 (Jun 2018 – Nov 2020) - R019 | 2315 | 14069.7 | 16.45(15.79-17.13) |
| **Total** |  |  |  |

*‡ The median date represents the midpoint of the survey visit period.*

*‡ Phone number change was defined as a participant providing a completely new set of numbers at a subsequent visit. Participants with multiple numbers were considered to have changed their phone contact only if all numbers had changed, placing them at risk of loss to follow-up in phone-based surveys.*

The incidence of mobile phone number change ranged between 15.7-19.1/100 person years and consistent across the 10 years of observation

**Table S2: Change of line for multiple line holders who had a follow up visit**

|  | **Changed line for multiple line holders** | | | |
| --- | --- | --- | --- | --- |
| **Survey median date (range) - visit number** | Never changed line | Changed only one line | Changed all the lines | Total |
| June 2012 (Aug 2011 – May 2013) - R015 | 0(0) | 20(36.4) | 35(63.6) | 55 |
| Jul 2014 (Jul 2013 – Jan 2015) - R016 | 4(2.6) | 94(60.6) | 57(36.8) | 155 |
| Jan 2016 (Jan 2015 – Sept 2016) - R017 | 31(13) | 182(76.2) | 26(10.9) | 239 |
| Jul 2017 (Oct 2016 – May 2018) – R018 | 53(13.5) | 290(73.8) | 50(12.7) | 393 |
| Aug 2019 (Jun 2018 – Nov 2020) - R019 | 65(14.9) | 321(73.5) | 51(11.7) | 437 |
| Total | 153(12) | 907(70.9) | 219(17.1) | 1279 |

*‡ Participant was considered to have changed all their lines if none of the previously listed phone numbers were provided at the subsequent visits. If a participant added an additional line to previously existing phone number(s), that was not considered a change.*

Among those who reported possession of more than one mobile phone number, the rate of change of at least one of the numbers at the subsequent visit(s) was over 70% for most of the survey visits,
